# Effect of Calcium Oxide on Stress Crack Resistance and Light Transmittance in PET Containers for Packaging Carbonated Beverages

**DOI:** 10.1021/acsomega.3c07193

**Published:** 2024-01-08

**Authors:** Gökçen Yuvalı, Esen Dagasan Bulucu, Bilal Demirel, Ali Yaraş, Fatih Akkurt, Sedat Sürdem, Burçak Demirel

**Affiliations:** † Department of Pharmaceutical Biotechnology, Erciyes University, Kayseri 38280, Turkey; ‡ Department of Material Science and Engineering, Erciyes University, Kayseri 38030, Turkey; § Department of Metallurgy and Materials Engineering, Bartin University, Bartin 74110, Turkey; ∥ Department of Chemical Engineering, Gazi University, Ankara 06560, Turkey; ⊥ Graduate School of Natural and Applied Sciences, Gazi University, Ankara 06500, Turkey; # Department of Electrical–Electronics Engineering, Abdullah Gul University, 38080 Kayseri, Turkey

## Abstract

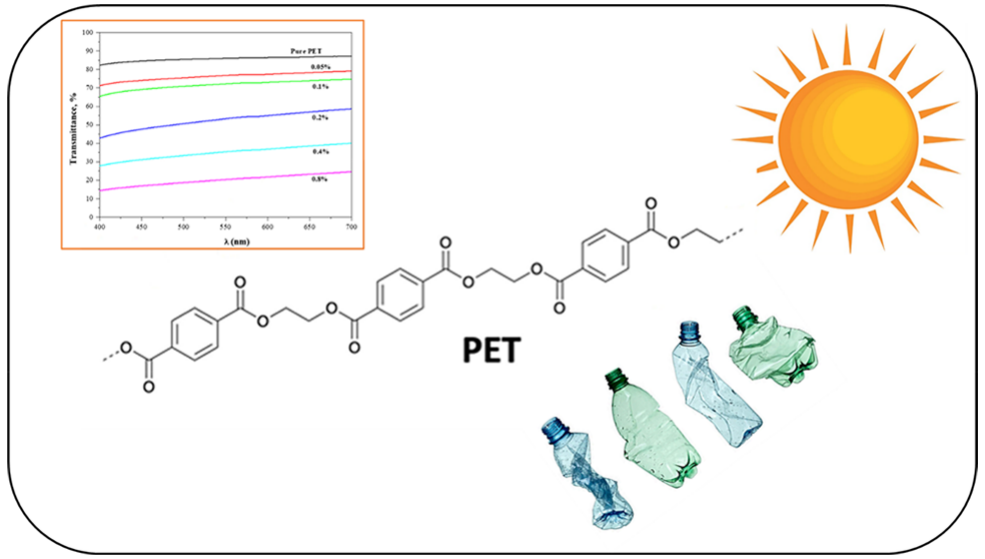

For polyethylene terephthalate (PET) bottles, a material used for food packaging, light transmission and mechanical performance, particularly environmental stress cracking (ESC), are essential characteristics. For this purpose, following extrusion of PET/CaO granules, preforms were manufactured using the injection technique, and bottles were produced by a stretch–blow–molding process. With incorporation of calcium oxide (CaO), light transmittance increased by around 25%, and ESC went from 0.3 to 11 min. In addition, whereas acetaldehyde (AA) and carboxylic acid (COOH) decomposition values rose with increasing CaO content, diethylene glycol and isophthalic acid values did not significantly change. Moreover, the maximum crystallization temperature and crystallinity both exhibited an upward trend with the CaO content.

## 1 Introduction

Polyethylene terephthalate (PET) is a type of transparent, high-strength, and chemically stable polymer that belongs to the thermoplastic polyester family. It is a synthetic polymer that is most frequently utilized in the food and beverage packaging industry.^1,2^ PET holds about 67% of the market, particularly in the beverage industry (water, carbonated soft drinks, tea, and coffee).^3^ PET material for these drinks should be chosen with filling/transport temperatures, internal compressive strength, and preservation of the quality of the food throughout the shelf life in mind. There are three types of beverage packaging: carbonated soft drinks, hot fillings, and cold filling. Water and other cold-filled items do not require sterilization (high temperature). The hot filling procedure is used to package products, such as tea and isotonic drinks. For carbonated beverages, it is desirable to use packages that can withstand 5 bar of internal pressure at room temperature. The characteristics of PET packaging materials are critical both for preservation of taste, smell, and nutritional values of foods and for consumer health and safety.^4,5^ In addition to the aforementioned requirements, research studies are also ongoing to enhance UV permeability, gas barrier, and chemical migration properties of PET composites.^6−9^ For instance, to stop food in PET bottles exposed to sunlight from degrading as a result of a photochemical reaction, several additives are added to the PET body.^10,11^

Environmental stress cracking (ESC) of PET bottles, a fragile sort of damage that slowly progresses, is one of the mechanical performance criteria. It is commonly known as plastic killer and responsible for at least 15% of plastic damage.^12^ The characteristics of the fluid with which the material comes into contact, applied tensile force, and kind of material all affect ESC phenomena, also known as crack development and propagation.^13^ Even though these factors make the event complex, ESC behavior is primarily influenced by applied force and liquid substance to which the material is exposed. As a result of tensile stress, the liquid first enters the polymer body’s free volume, causing localized swelling. Next, the chain mobility starts. As the locally plasticized material’s yield strength declines, cracks start to form where the material and liquid come into contact, resulting in mechanical damage.^14^ Load-carrying capacity is another mechanical durability requirement that is looked for during the manufacture, distribution, and storage of PET bottles. As a result, it is inevitable that different measurement techniques be used to describe the mechanical performance of the PET bottle.

In the context of this investigation, PET/CaO granules were created by extrusion, preforms were produced by injection, and bottles were produced using a blow–stretch molding technique. The study provides significant results in terms of mechanical performance (particularly environmental cracking resistance), which is crucial for a plastic bottle, and light transmittance. Additionally, the final PET bottles were analyzed for chemical migration and thermal properties.

## 2 Materials and Methods

CaO of analytical purity was purchased from Kimetsan Kimya. PET with a 0.84 dL/g intrinsic viscosity and 1 ppm (maximum) acetic acid (AA) content was supplied in granule form from Köksan Plastik packaging company (Gaziantep/Turkey).

PET granules and CaO powders were dried at 80 °C for 24 h before extrusion. PET/CaO composites were melted and blended at a 100 rpm screw rotation speed in a corotating twin screw extruder (Gülnar Makina, Turkey) with a length/diameter ratio of 40:1. Extruder temperatures from the feed zone to the nozzle were set at 50, 200, 205, 210, 215, and 220 °C. The dried CaO powders were fed into the extruder together with PET granules in five different amounts (0.05, 0.1, 0.2, 0.4, and 0.8% by mass). PET/CaO composites were taken from the extruder as granules and stored in a closed container for use in the injection process.

After drying PET/CaO granules for 12 h at 80 °C, they were fed into a vertical injection device (YH-15 V, Yuhdak Makina, Taiwan) and injected into a preform mold for bottle production. The mold temperature of the vertical injection device was set at 15 °C, and barrel regional temperatures were set as 260, 275, and 280 °C. The stretch–blow–molding method was applied to the produced preforms for bottle production.

**Figure 1:**
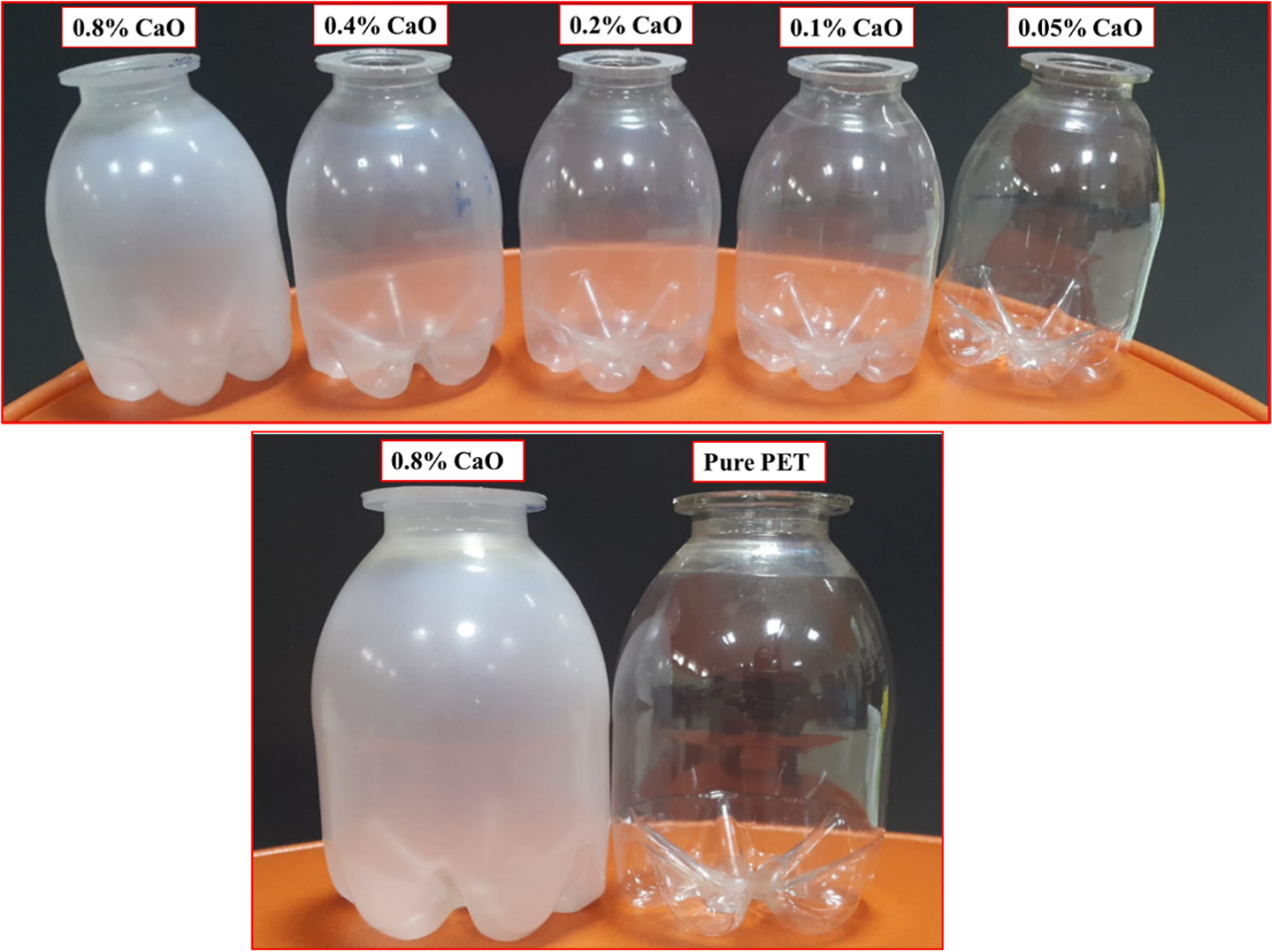
Digital images of PET bottles produced.

In the semiautomatic SBM machine, the heating process was provided by eight IR lamps. In the SBM process, the surface temperature of the preform varies between 90 and 95 °C. During stretching and blowing, pressure was held at 0.09 MPa for 0.5 s. Then, pressure was increased to 1.5 MPa in 2 s and held for 2 s at a1.5 MPa pressure. The speed of the tension bar was 0.75 m/s, reaching the bottom of bottle in 1.13 s. During the blowing process, mold temperature and residence time were set at 20 °C and 2 s, respectively. The digital images of PET bottles produced are presented in Figure 1.

### 2.1 Characterization and Analyses

The particle size of CaO was determined using a particle size distribution analyzer (Malvern, Nano ZS90). Fourier transform infrared spectroscopy (FTIR) analyses (PerkinElmer 400) of pure PET and PET/CaO composites were performed at a wavelength range of 4000–400 cm^–1^, at a resolution of 4 cm^–1^, and by averaging 20 scans. Phase structures of CaO, PET, and PET/CaO composites were determined by X-ray diffractometry (XRD) analyzes (Bruker AXS D8) performed between 2θ = 5 and 90°. Surface morphological features of the samples were also characterized by scanning electron microscopy (SEM) analysis (ZEISS LS-10).

Thermal properties of specimens were determined by differential scanning calorimetry (DSC) (PerkinElmer Diamond) and thermogravimetric analysis (TGA) (Hitachi-High TechSTA-7300) analyses. DSC and TGA analyses were performed at a heating rate of 10 °C/min and in a nitrogen atmosphere.

Chemical degradation analyses of AA, COOH, isophthalic acid (IPA) and diethylene glycol (DEG) were performed according to F2013-01, DIN/ISO 2114, IQ10694, and IQ10687 standards, respectively. For COOH analysis, 4 g and 50 mL (1:1 phenol/chloroform) were taken into a beaker and refluxed for 1 h until a clear solution was obtained. Then, 0.5 mL of 0.1% ethanolic tetrabromophenol blue solution was added to the solution at room temperature and mixed. The prepared solution was titrated with 0.1 N benzylalcoholic KOH solution until the color changed from yellow to blue.

For AA analysis, PET/CaO samples were cooled with liquid nitrogen, ground, and passed through a 750 μm sieve. Waiting for about 10 min allowed the samples to reach room temperature. 0.2 g of the ground sample was placed in the headspace, and the amount of AA was analyzed with gas chromatography.

According to the analysis procedure of IPA and DEG, the ground sample was placed in a sealed container of 50 mL, and then 10 mL of standard solution and 1 mg of zinc acetate were added. The container was left in an oven at 220 °C for 2 h. After the temperature of the container reached ambient temperature, 15 mL of chloroform was added to it. The sample was filtered through a membrane filter; the filtrate was taken into a headspace and analyzed with gas chromatography. Analyses were performed in triplicate, and mean values were used in calculations.

Mechanical tests were carried out using a tensile testing device (DVT GP E NN) at a speed of 100 mm/min according to the EN ISO 527-3 standard. The tensile test was repeated three times for each sample, and average values were taken.

## 3 Results and Discussion

### 3.1 XRD and FTIR Analyses

**Figure 2:**
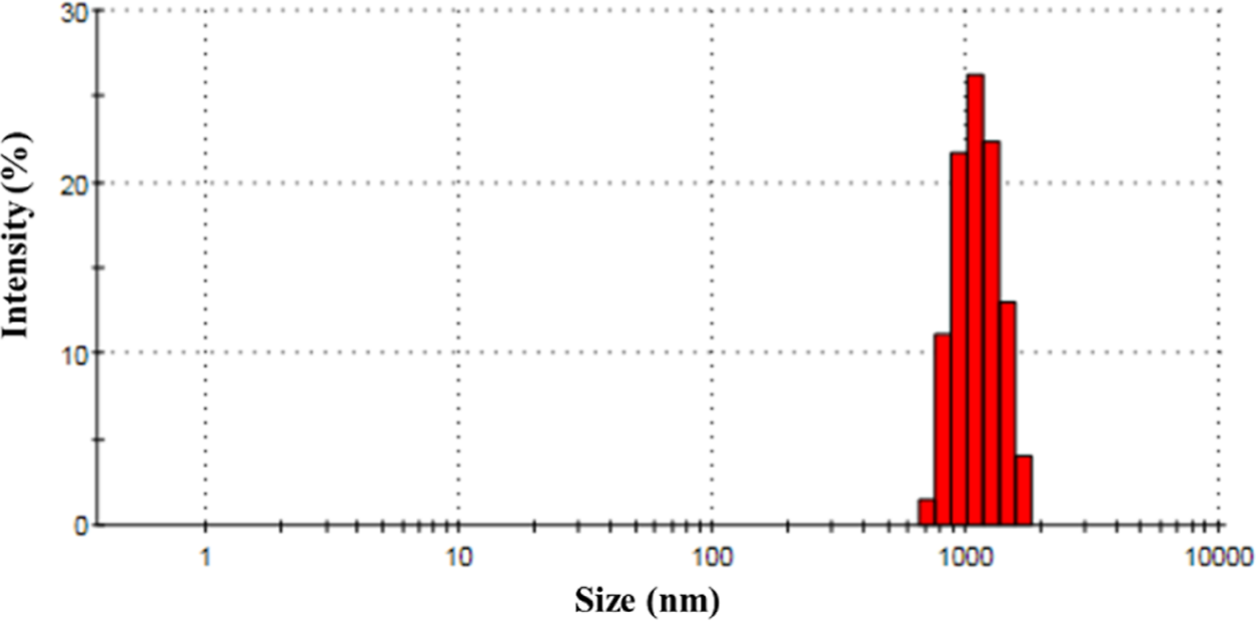
Particle size analysis distribution of CaO.

**Figure 3:**
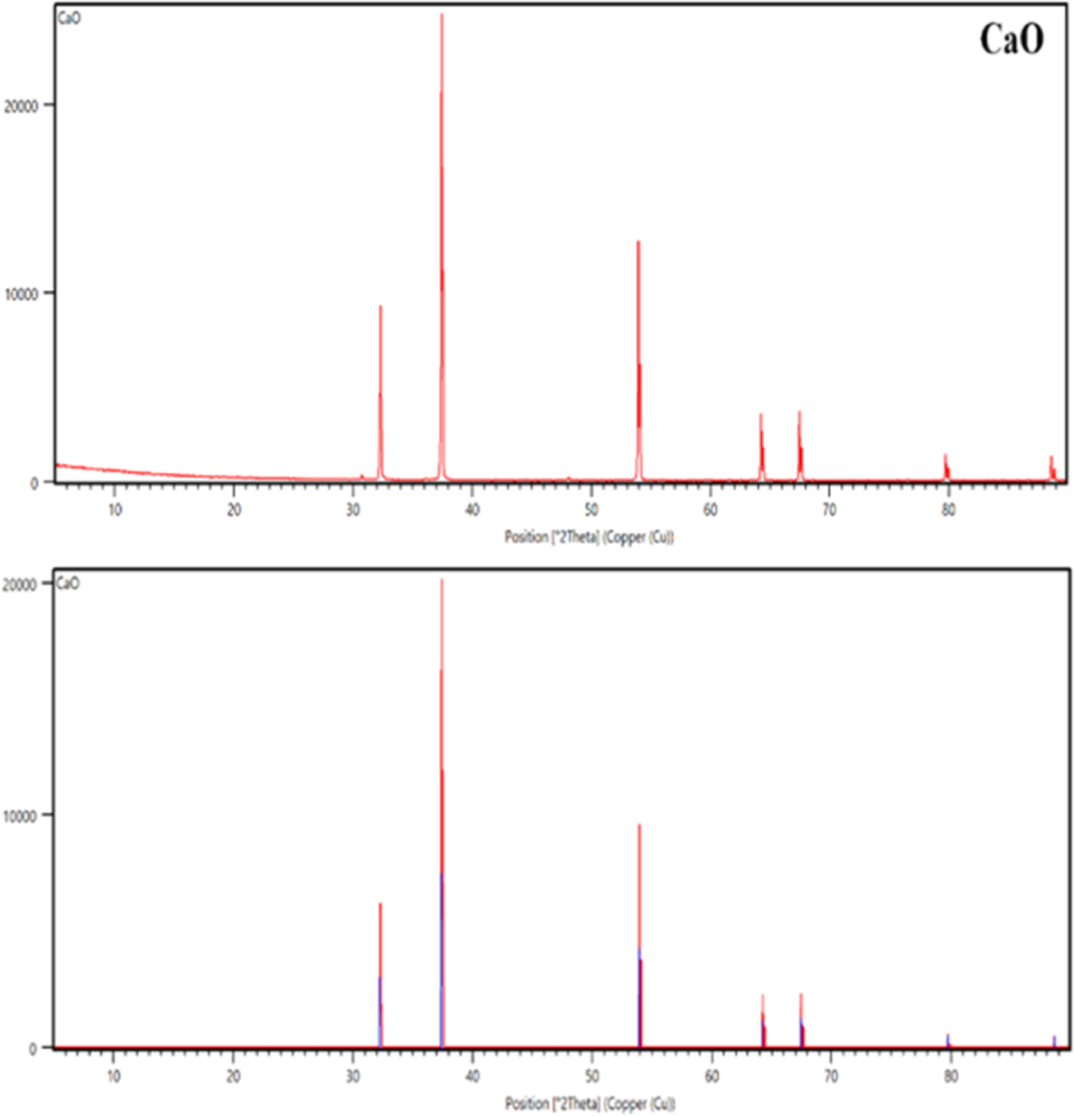
XRD pattern of the CaO particle.

In Figure 2, a size distribution analysis of CaO particles utilized in experiments is displayed. As a result, CaO particles have an average particle size of 1149 nm. Figure 3 displays the crystalline phases of the CaO particle. Accordingly, the diffraction peaks in the range of 10–90° overlap with the values of previous researchers (reference code: 01-077-2010).^15−17^ The major peak at 2*Q* = 37.4 (200) and the peaks appearing at 32.3, 37.4, 54.0, 64.1, 67.5, 79.8, and 88.6° are correlated with the planes (111), (200), (220), (311), (222), (400), and (331), respectively.^15^

**Figure 4:**
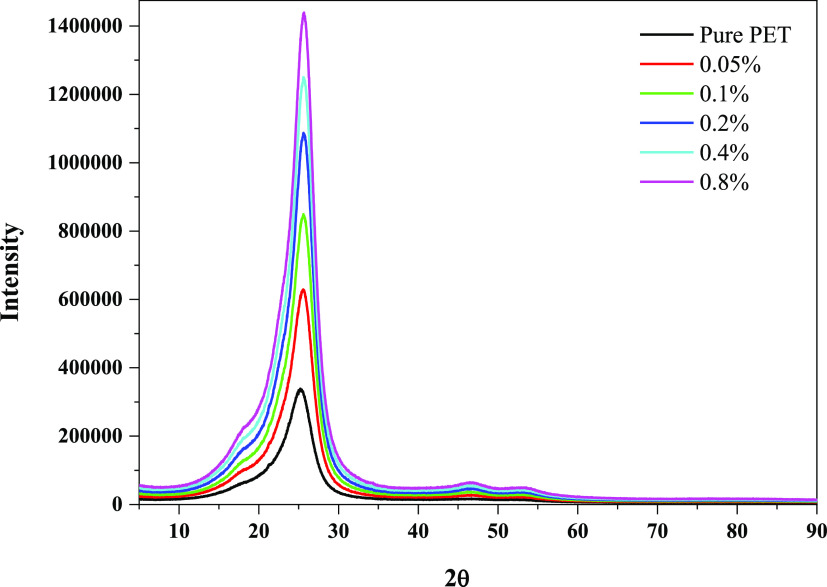
X-ray patterns of PET specimens with/without CaO.

It is well-known that the typical PET diffraction peak occurs at approximately 2*Q* = 26.6°.^18,19^ With the incorporation of CaO, the peak intensity of the PET composite increased, but the 2*Q* value did not change (Figure 4). The reason for this may be that because so little amount CaO is used, PET suppresses the calcium oxide peaks. All these findings suggest that CaO and the PET matrix do not interact chemically.

**Figure 5:**
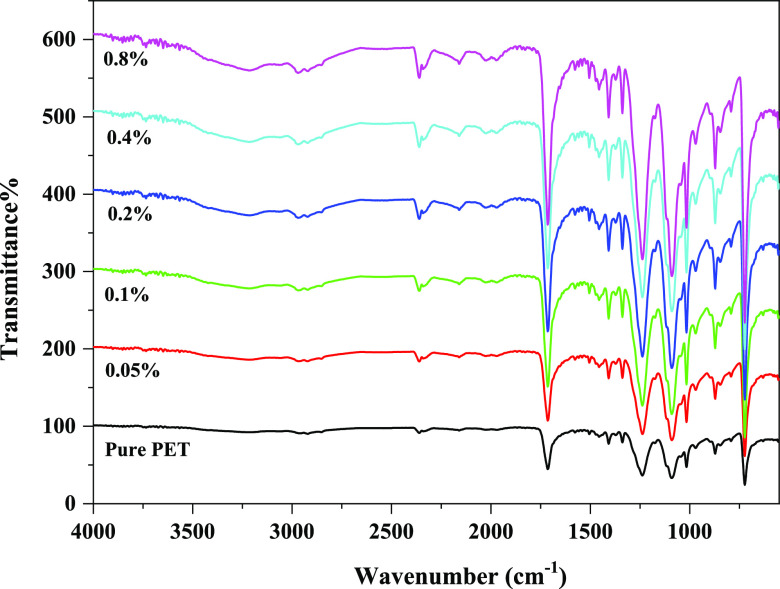
FTIR spectra of pure PET and PET/CaO composites.

Aliphatic and aromatic C–H stretches in the PET chain are seen in the IR spectra at 2800 and 3100 cm^–1^, respectively (Figure 5). Esteric bonds at approximately 1710 and peaks at 1300 cm^–1^ are responsible for C–O symmetric stretching of the carbonyl group and vibrations of ester groups. In addition, IR spectra at 1165 and 1100 cm^–1^ are associated with C–O–C asymmetric stretching vibrations and methylene group stretching, respectively.^20^ On the other hand, the FTIR spectrum around 3650 cm^–1^ originates from the O–H band of the water molecule on the surface of the CaO particle.^21^ It belongs to CaOH, and it becomes more broad as the CaO content increases. The band at about 2350 cm^–1^ is due to the presence of atmospheric CO_2_.^22^ The main characteristic peak of CaO is the vibration of the Ca–O bond at 557 cm^–1^.^23^

### 3.2 Chemical Degradation Analysis

The sensory qualities of the product in the bottle could be negatively impacted by acetaldehyde in the PET structure. Because AA has a high solubility in water, it can easily pass to foods with high water content. As a result, due to the risk of acetaldehyde migration, PET bottles used for beverage packaging have become more significant. According to findings in Table 1, AA migration is higher in all CaO-incorporated samples than pure PET. The AA migration value reached 20.25 ppm as the CaO content rose. This rise can be due to CaO’s strong polar nature and high electron affinity, which enable it to interact with hydrogen ions.

**Table 1:** Chemical Degradation Results

CaO, %	DEG, %	IPA, %	AA, ppm	COOH (mmol/kg)
0	1.52 ± 0.041	1.81 ± 0.0202	6.36 ± 0.865	60.05 ± 0.777
0.05	1.82 ± 0.048	1.82 ± 0.0137	11.93 ± 0.786	65.73 ± 0.869
0.1	1.84 ± 0.053	1.79 ± 0.0168	12.38 ± 0.821	68.58 ± 0.811
0.2	1.85 ± 0.056	1.81 ± 0.0179	15.05 ± 0.783	73.94 ± 0.712
0.4	1.88 ± 0.052	1.80 ± 0.0171	18.73 ± 0.837	75.91 ± 0.874
0.8	1.88 ± 0.054	1.80 ± 0.0183	20.25 ± 0.859	82.91 ± 0.732

CaO exhibits a catalytic effect on the degradation of COOH in PET. This can be explained by the fact that the hydrolysis reaction accelerates the degradation of COOH.^24^ In other words, the greater the presence of COOH groups in the PET matrix, the higher the moisture holding capacity of the polymer because water’s hydrogen bonds and carboxyl groups make PET more affine to water and facilitate the hydrolysis reaction.^25^ In this work, the incorporation of CaO resulted in PET chains that were shorter and had more COOH decomposition. Similarly, previous investigations have shown that usage of inorganic additives CaB_2_O_4_^8^ and Ca_3_B_2_O_6_^26^ promoted COOH degradation. On the contrary, COOH degradation tended to decrease in a study when Mg_2_B_2_O_5_ was added to PET. The affinity of the B2O3 anion to COOH is thought to be the cause of this.^27^

While DEG revealed a relative increase for all samples, IPA values did not differ from pure PET in comparison.

### 3.3 Thermal Analysis

**Figure 6:**
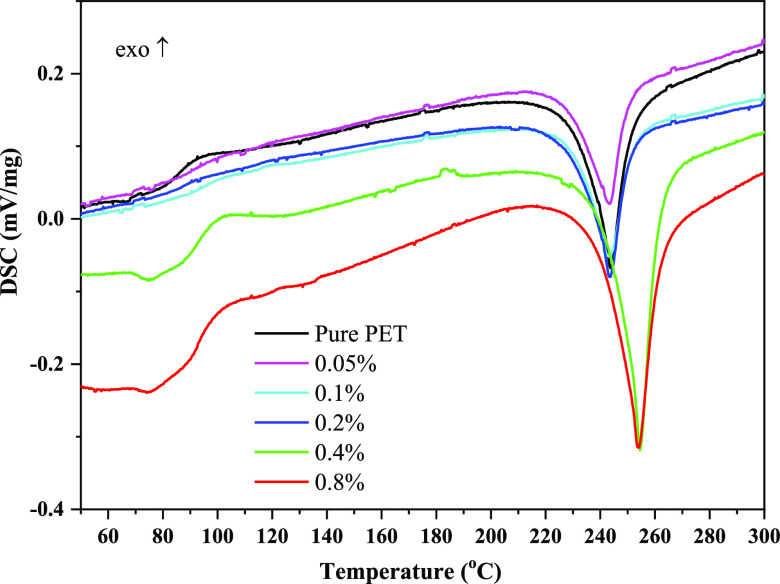
DSC curves of PET samples with/without CaO.

Table 2 demonstrates that both the melting temperature (*T*_m_) and glass-transition temperature (*T*_g_) did not vary noticeably (Figure 6). Also, it is obvious that adding CaO promotes crystallinity (*X*_c_). Comparing this rise to pure PET, it is around 57% higher. This might be as a result of uniform dispersion of CaO particles throughout the PET matrix.^28^ On the other hand, it has been noted that as particles clump together within the polymer structure, particle nucleation and chain mobility are restricted, connection between chains becomes weaker, and as a result, crystallinity falls.^29^ The crystallization temperature rose, as well. This may be attributed to the interaction of calcium oxide with high affinity with H^+^ ions in PET and reduced chain mobility.

**Table 2:** TG/DTG Properties of Produced PET Bottles^a^

CaO, %	*T*_m_, °C	*T*_g_, °C	*T*_c_, °C	*X*_c_, %
0	249.3	81.2	188.4	17.8
0.05	249.1	81.1	191.8	18.9
0.1	249.2	81.2	193.4	21.6
0.2	249.3	80.9	195.2	24.3
0.4	249.5	80.8	198.2	26.9
0.8	249.7	80.9	200.8	28.0

a *T*_m_: melting temperature, *T*_g_: glass transition temperature, *T*_c_: crystallization temperature, *X*_c_: percent crystallinity.

### 3.4 Mechanical Tests

Carrying PET bottles on top of each other may cause breakage in the side or bottom areas. This entails both the loss of the food item and transportation-related stacking issue. Therefore, load-carrying strength is crucial for PET bottle, transport, and stacking/storage procedures. It is obvious that the load-carrying capacity increases with the incorporation of CaO (Table 3). With a rise of around 115.1% over the reference value (86.5 N), the highest (186.1 N) was attained in the presence of 0.8% CaO. The reason for this increase is thought to be crystallinity. In other words, the stiffness rose and mobility of polymer chain reduced with the incorporation of CaO.^30,31^

**Table 3:** Mechanical Performance Results of PET Bottles Produced

CaO, %	load-carrying capacity, N	shrinkage, %	ESC, min	burst strength, MPa
0	86.50	26.3	0.3	6.59
0.05	144.0	26.9	44	11.81
0.1	151.5	27.5	28	11.66
0.2	153.9	28.9	26	11.60
0.4	167.2	29.7	18	11.07
0.8	186.1	30.3	11	10.81

Prolonged exposure of bottles to high temperatures, resulting in dimensional deformations (shrinkage), is a concern, especially for hot fill packages. According to data in Table 3, the shrinkage ratio of bottles increased as CaO was incorporated. This rise rate is at its highest level of 15.2% when compared to the CaO-free bottle. Shrinkage occurs mainly in the body region of bottles. Consequently, it is important to consider bottles’ unit weight (weight/area). As a matter of fact, more material was moved to the base and shoulders than the body throughout the stretching process (Table 4).

**Table 4:** Weight Distribution of Produced PET Bottles

CaO, %	shoulder, g	body, g	base, g
0	5.8	1.3	2.3
0.05	5.4	1.2	2.7
0.1	5.2	1.2	2.5
0.2	5.4	1.2	2.5
0.4	5.3	1.2	2.6
0.8	5.5	1.2	2.6

ESC is defined as crack formation and propagation that occurs depending on various parameters such as material type, the applied force, and fluid with which the material is in contact.^13,32^ However, ESC behavior is basically caused by the dual impact of tensile stress applied to the material and the liquid agent to which the material is subjected.^33^ This study used NaOH solution (2%) as the chemical liquid agent for the ESC test, which was conducted under a 2 bar load.^34^ CaO-free PET sample’s ESC value was determined to be 0.3 min. It displayed a declining trend after 44 to 11 min, rising from 0.05 to 0.8% CaO content. In conclusion, ESC values for all additive ratios were much greater than for pure PET, as shown in Table 3.

Burst strength is the pressure at which the bottles rupture at their weakest point following the application of an internal pressure (carbonation). This pressure is crucial for measuring the amount of filling in postproduction filling of carbonated beverages and for keeping an eye on excessive expansion of bottles after filling that results from heating. In comparison to pure PET, the burst strength rose with the incorporation of 0.05% CaO, but no discernible difference was seen at higher CaO contents (Table 3). The uniform distribution of CaO particles and transmission of external load to CaO particles inside the polymer matrix may be the cause of this rise.^35,36^

### 3.5 SEM Analysis

**Figure 7:**
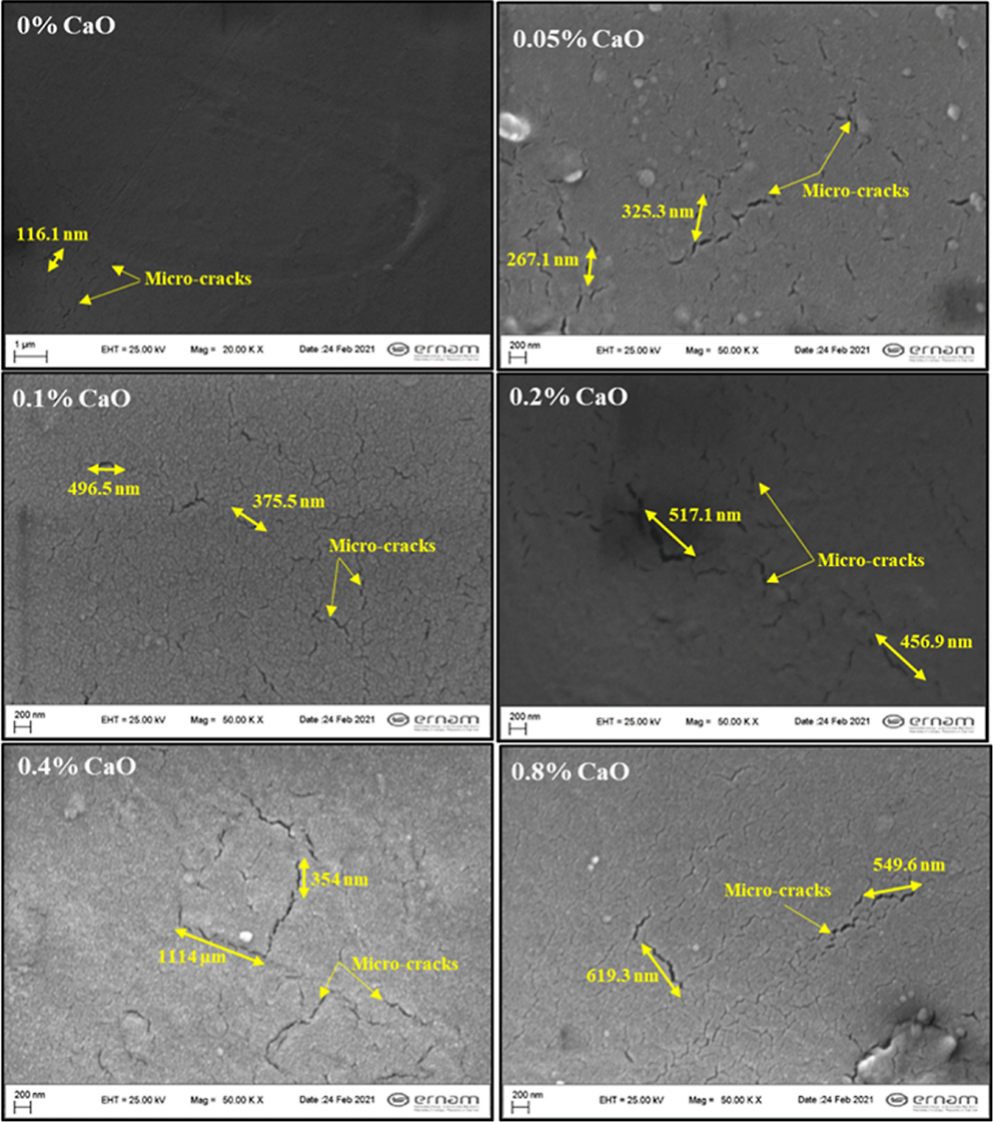
SEM images of PET samples with/without CaO.

Figure 7 displays SEM images of PET bottles with and without CaO. In all samples with additives, in addition to pure PET, it is notable to find microcracks of various lengths. Undoubtedly, having cracks in a packaging material is unfavorable because they allow for gas penetration. In the polymer matrix, CaO particles also showed a uniform dispersion.

### 3.6 Optical Properties

**Figure 8:**
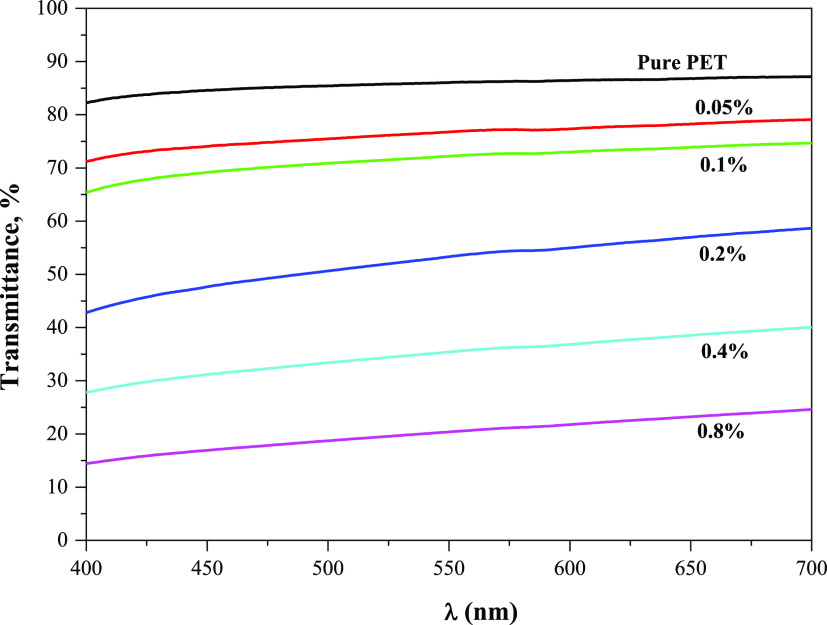
Results of UV light transmittance testing on PET bottles with/without CaO.

According to the findings in Figure 8, pure PET transmits light at a rate of more than 80%. It is also obvious that the light transmittance decreases with increasing CaO content. UV transmittance did not significantly change with the addition of 0.05 and 0.1% CaO, remaining at levels of roughly 75 and 70%, respectively. Light transmittance dropped to 50% in the presence of 0.2% CaO, and this value was measured as 20% with a 0.8% CaO content. These findings, which apply to both doped PETs and pure PET, are accurate in the visible light spectrum. The high light-absorbing capacity of CaO may be the cause of this.^37^ Researchers claim that inorganic additions like ZnO,^38^ TiO_2_,^39^ and hydroxyapatite nanoparticles^40^ diminish the light transmission of PET. Furthermore, a similar trend was observed in terms of UV transmittance for calcium-containing boron compounds (CaB_2_O_4_, Ca_3_B_2_O_6_).^8,26^

## 4 Conclusions

This study highlighted the impact of CaO on mechanical (particularly ESC test), chemical degradation, and optical characteristics of PET bottles. Experimental results can be summed as follows;

- Degradation of DEG and IPA was unaffected by the presence of CaO; however, AA and COOH degradation were accelerated.
- Load carrying capacity and burst strength both improved with the incorporation of CaO. PET/CaO composites degraded more slowly than pure PET even under challenging conditions (2% NaOH solution and 2 bar pressure).
- Crystallinity and crystallization temperature both increased in response to the addition of CaO.
- With a 25% reduction in UV transmittance, there is now a chance to lessen photocatalytic deterioration of food in PET bottles.

Our future research is intended to investigate the use of various organic and inorganic additives to prevent PET cracks, which lead to gas permeability and subsequently food deterioration.
